# Development of a rapid detection method for *Karenia mikimotoi* by using CRISPR-Cas12a

**DOI:** 10.3389/fmicb.2023.1205765

**Published:** 2023-08-07

**Authors:** Lu Wang, Xiaoyao Chen, Feifei Pan, Guangshan Yao, Jianming Chen

**Affiliations:** ^1^Fujian Key Laboratory on Conservation and Sustainable Utilization of Marine Biodiversity, Fuzhou Institute of Oceanography, Minjiang University, Fuzhou, China; ^2^Fishery Resources Monitoring Center of Fujian Province, Fuzhou, China

**Keywords:** *Karenia mikimotoi*, harmful algal bloom, LbCas12a, internal transcribed spacer, lateral flow dipstick assay, recombinase polymerase amplification

## Abstract

Harmful algal blooms (HABs), mainly formed by dinoflagellates, have detrimental effects on marine ecosystems and public health. Therefore, detecting HABs is crucial for early warning and prevention of HABs as well as the mitigation of their adverse effects. Although various methods, such as light microscopy, electron microscopy, real-time PCR, and microarrays, have already been established for the detection of HABs, they are still cumbersome to be exploited in the field. Therefore, rapid nucleic detection methods such as recombinase polymerase amplification (RPA) and loop-mediated isothermal amplification (LAMP)-lateral flow dipstick (LFD) have been developed for monitoring bloom-forming algae. However, the CRISPR/Cas-based detection of HABs has yet to be applied to this field. In this study, we developed a method for detecting *Karenia mikimotoi* (*K. mikimotoi*), a typical ichthyotoxic dinoflagellate responsible for global blooms. Our method utilized Cas12a from Lachnospiraceae bacterium ND2006 (LbCas12a) to target and cleave the internal transcribed spacer (ITS) of *K. mikimotoi*, guided by RNA. We leveraged the target-activated non-specific single-stranded deoxyribonuclease cleavage activity of LbCas12a to generate signals that can be detected using fluorescence-read machines or LFDs. By combining RPA and LbCas12a with reporters, we significantly enhanced the sensitivity, enabling the detection of ITS-harboring plasmids at concentrations as low as 9.8 aM and genomic DNA of *K. mikimotoi* at levels as low as 3.6 × 10^−5^ ng/μl. Moreover, we simplified the genomic DNA extraction method using cellulose filter paper (CFP) by directly eluting the DNA into RPA reactions, reducing the extraction time to < 30 s. The entire process, from genomic DNA extraction to result reporting, takes less than an hour, enabling the identification of nearly a single cell. In conclusion, our method provided an easy, specific, and sensitive approach for detecting *K. mikimotoi*, offering the potential for efficient monitoring and management of *K. mikimotoi* blooms.

## 1. Introduction

Harmful algal blooms (HABs) are formed by more than 200 species of algae, including Cyanophyta, Cryptophyta, dinoflagellates, and diatoms (Sournia, 1995). These bloom-forming species have detrimental impacts on marine environments, human health, and the economy through toxin production or biomass accumulation (Glibert et al., 2005; Paerl et al., 2011; Grattan et al., 2016). Dinoflagellates and diatoms are significant contributors to the harmful marine algae associated with HABs (Duran-Vinet et al., 2021).

*Karenia mikimotoi* (*K. mikimotoi*) is one of the species of dinoflagellates known for forming HABs. It was initially identified by Dr. Shoichi Miyake and Konan Kiyo in Japan in 1935 (Oda, 1935) and has been observed in coastal waters worldwide, including China (Li et al., 2017, 2019b). This species exhibits recurrent blooms, particularly in the East China Sea, leading to significant mortality of fish and shellfish populations (Li et al., 2017). The blooms that occurred in the coastal waters of Fujian province in 2012 attracted significant attention due to its devastating impact on farmed abalone, resulting in economic losses exceeding US$330 million (Li et al., 2017). Considering the detrimental effects and economic consequences, an early warning system for *K. mikimotoi-*formed bloom is urgently required.

Various methods have been developed for the detection of HABs, ranging from traditional light microscopy to molecular methods (Duran-Vinet et al., 2021). Morphological and molecular methods are commonly employed for HAB monitoring. Light microscopy is widely used for morphological identification and cell enumeration. The Moderate Resolution Imaging Spectroradiometer (MODIS) (Siswanto et al., 2013; Kurekin et al., 2014) and spectral light absorption (Stæhr and Cullen, 2003) have been applied to monitor *K. mikimotoi*. However, these conventional methods are ineffective and time-consuming and require taxonomic expertise (Toldrà et al., 2020). In response to these challenges, nucleic acid-based molecular methods have been employed for HAB monitoring, including *K. mikimotoi*. The semiautomated sandwich hybridization assay targeting large subunits of rRNA has been developed as a nucleic acid detection method (Haywood et al., 2007), but its sensitivity needs improvement. To enhance the sensitivity, pre-amplification is commonly employed. Polymerase chain reaction (PCR) is a frequently used nucleic acid amplification method, which involves multiple thermal cycles for initialization, denaturation, annealing, extension, and final elongation. Quantitative polymerase chain reaction (qPCR) (Yuan et al., 2012; Hu et al., 2022), conventional PCR-based detection (Guillou et al., 2002; Al-Kandari et al., 2011), and real-time nucleic acid sequence-based amplification with internal control RNA (IC-NASBA) (Ulrich et al., 2010) have been utilized for monitoring *K. mikimotoi*, often coupled with fluorophore and quencher, or electrophoresis for detection. However, the field application of these PCR-based methods is limited by their dependence on instrument-based thermal cycling. To overcome this limitation, isothermal amplification techniques that do not require thermocyclers have shown promise for on-site application, including recombinase polymerase amplification (RPA) (Piepenburg et al., 2006), loop-mediated isothermal amplification (LAMP) (Notomi et al., 2000), and hyperbranched rolling circle amplification (Dean et al., 2001). Following pre-amplification, lateral flow dipstick (LFD) detection technology has been frequently used in conjunction with these isothermal amplification techniques for readout of the results, as demonstrated in methods developed for monitoring *K. mikimotoi*, such as hyperbranched rolling circle amplification coupled with LFD (Zhang et al., 2019) and LAMP-LFD (Huang et al., 2020; Wang et al., 2020; Han et al., 2022).

In recent years, clustered regularly interspaced short palindromic repeats/Cas proteins (CRISPR/Cas)-based molecular detection systems have been proposed as next-generation biomonitoring tools for HABs (Duran-Vinet et al., 2021). CRISPR/Cas is an adaptive immune system found in bacteria and archaea, consisting of CRISPR arrays and Cas nucleases (Doudna and Charpentier, 2014). It has been widely employed in genetic engineering and is classified into two classes (Class 1 and Class 2), with six types (Type I to Type VI) in each class (Xu and Li, 2020). Beyond its genome editing capabilities, the collateral activity of two Class 2 Cas proteins, namely Cas13 and Cas12, makes this system a promising diagnostic tool for the next generation (Chen et al., 2018; Kellner et al., 2019, 2020). Cas12a, formerly known as Cpf1 (Zetsche et al., 2015), belongs to class 2, type V CRISPR/Cas system. The Cas12 enzymes, which include AsCas12a (Acidaminococcus sp.BV3L6), FnCas12a (*Francisella novicida* U112), LbCas12a (Lachnospiraceae bacterium ND2006), and MbCas12a (Moraxella bovoculi 237), cleave target DNA by recognizing a 5' T-rich protospacer-adjacent motif (PAM) using a guide RNA without trans-activating CRISPR RNA and generate PAM-distal staggered 5' and 3' ends (Zetsche et al., 2015). In addition to its genome editing capabilities, Cas12a has been found to possess “collateral activity” on single-stranded DNAs (ssDNAs), leading to its utilization in diagnostic platforms with pre-amplification, such as DNA endonuclease-targeted CRISPR trans reporter (DETECTR) (Chen et al., 2018), a 1-h low-cost multipurpose highly efficient system (HOLMES) (Li et al., 2018), and HOLMESv2 (Li et al., 2019a). Since then, Cas12a-based platforms have been extensively employed for detecting pathogenic bacteria, viruses, and food contaminants (Yang et al., 2023) but have yet to be applied for HAB detection and monitoring (Duran-Vinet et al., 2021).

**Figure 1:**
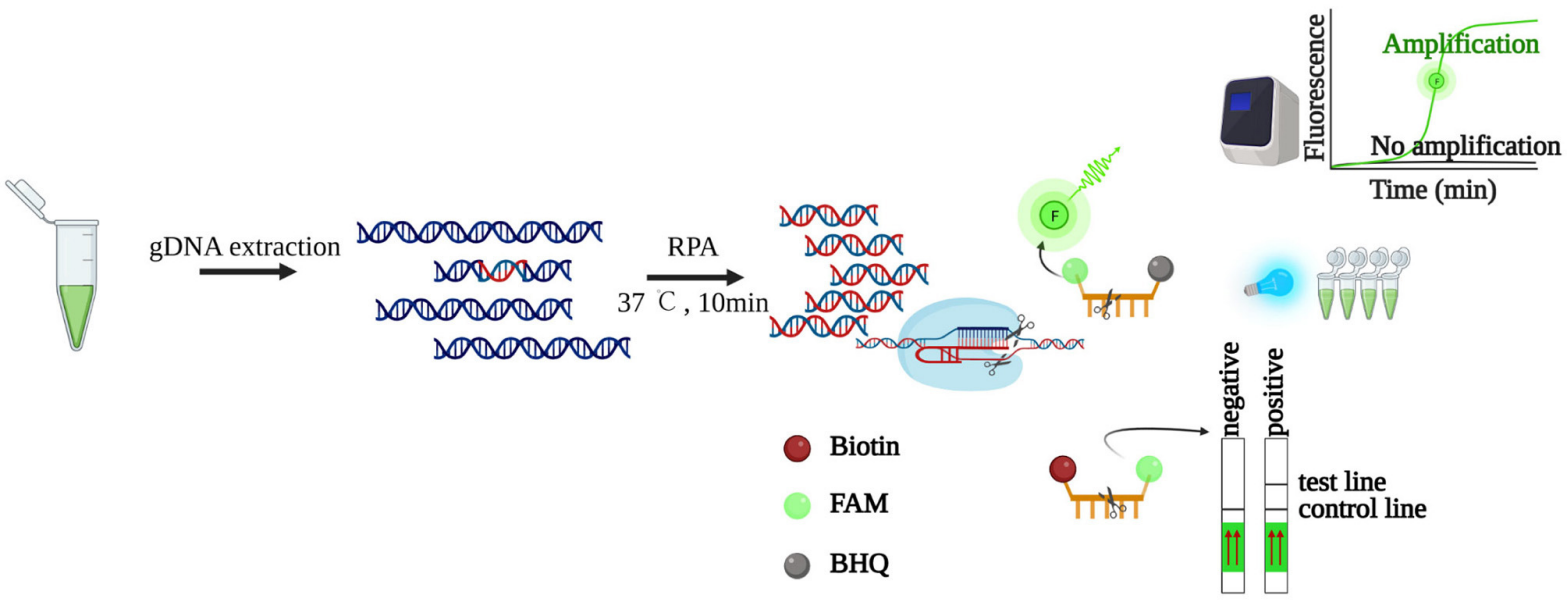
Schematic illustration of LbCas12a-based detection. The genomic DNA of *K. mikimotoi* is extracted, followed by RPA pre-amplification. By identifying and digesting specific target DNA, the “collateral activity” of LbCas12a is activated, which will digest bystander non-specific ssDNAs. The released signals are detected by lateral flow strips or fluorescence readers such as a real-time PCR machine or blue light transilluminator. Created with BioRender (https://www.biorender.com/).

Here, for the first time, we present a CRISPR/LbCas12a-based method for detecting HAB-forming algae with *K. mikimotoi* as the representative species (Figure 1). By coupling the CRISPR/LbCas12a system with pre-amplification using RPA, we achieved an easy, sensitive, and specific platform for detecting and monitoring *K. mikimotoi*. Furthermore, we demonstrated the effectiveness of cellulose filter paper (CFP)-based DNA extraction (Zou et al., 2017) for algae genomic DNA extraction, enabling subsequent processes such as conventional PCR and RPA. Our developed method, combining CFP-based DNA extraction, RPA pre-amplification, and CRISPR/LbCas12a system, offers a cost-effective and field-deployable solution for detecting and monitoring the bloom-forming algae.

## 2. Materials and methods

### 2.1. Algal cultures and maintenance

As shown in Table 1, 10 cultures were used in this study and were originally isolated from the Southern or Eastern China Sea. Most of the cultures were provided by the Collection Center of Marine Bacteria and Algae at Xiamen University, China. All cultures were cultivated, as previously mentioned (Wang et al., 2014), in autoclaved 0.45 mm-filtered seawater (salinity 28PSU) amended with L1 medium nutrients. Illumination was provided by cool-white fluorescent light bulbs in a 12:12-h light: dark cycle with a photon flux of ~100 μmol photons m^−2^s^−1^. Temperature was maintained at 15 ± 1°C or 20 ± 1°C, depending on the natural environment where each strain was collected (Table 1).

**Table 1:** List of algae strains used in the study.

**Strain**	**Morphospecies designation**	**Origin**
CCMA444^a*^	*Heterocapsa* sp.	China: Pingtan Island
CCMA279^a^	*Amphidinium carterae*	China: The mouth of Yangtze River
WL25^b*^	*Prorocentrum triestinum*	China: Pingtan Island
CCMA79^a^	*Isochrysis galbana*	China: The mouth of Yangtze River
GY-H2^c^	*Dicrateria zhanjiangensis*	China
CCMA488^a*^	*Karlodinium veneficum*	China: Pingtan Island
CCMA37^a^	*Karenia mikimotoi* A	China: Shenzhen Bay
CCMA83^a*^	*Karenia mikimotoi* B	China: Su'ao Bay, Pingtan Island
CCMA607^a*^	*Karenia papilionacea*	China: Pingtan Island
CCMA61^a^	*Alexandrium pacificum*	South China Sea

^a^Culture source obtained from the Collection Center of Marine Bacteria and Algae, Xiamen University, China. ^b^Isolated by the author in this study. ^c^Bought from LeadingTec. ^*^Isolated from samples during bloom.

### 2.2. Regular genomic DNA extraction

Regular genomic DNA extraction was performed as previously described (Wang et al., 2014). In brief, algal cells in the exponential growth phase were collected by centrifugation at 4,500 × *g* for 10 min at 4°C. The cell pellets were then suspended in DNA lysis buffer (10 mM Tris pH 8.0, 100 mM EDTA pH 8.0, 0.5 % SDS, 100 μg/ml proteinase K) and incubated at 55°C for 2–3 days. DNA was extracted using cetyl/hexadecyl trimethyl ammonium bromide in 0.7 M NaCl solution method and purified using a DNA clean & concentrator column (Zymo Research, Orange, CA, USA). The final DNA samples were dissolved in 10 mM Tris-HCl buffer (pH 8.0), measured spectrophotometrically using NanoDrop D2000 (Thermo Fisher Scientific, Inc., Wilmington, DE, USA), and stored at 20°C.

### 2.3. CFP-based DNA extraction

The extraction was performed as previously described with a brief modification (Zou et al., 2017). For spiked samples, 5 × 10^5^ *K. mikimotoi* cells were collected and suspended in 50 ml of ocean water, followed by 10-fold gradient dilution (10^3^, 10^2^, 10^1^, 10^0^, 10^−1^, and 10^−2^ cells/ml) with ocean water. Subsequently, all types of samples were collected and resuspended with 500 μl lysis buffer (20 mM Tris-Cl, pH 8.0; 25 mM NaCl; 2.5 mM EDTA; and 0.05 % SDS), by handshaking with 0.5 mm ball bearings for 8 s in 1.5 ml Eppendorf tubes. A dipstick made of 2 × 40 mm wax-coated CFP containing a 2 × 4 mm nucleic acid-binding zone was dipped into the above lysate for 3 s, followed by a 3-s wash in wash buffer (10 mM Tris-Cl, pH 7.4; 150 mM NaCl; and 0.1 % Tween-20). Then, the bound DNA was directly eluted into the PCR or RPA reaction.

### 2.4. Internal transcribed spacer region amplification and cloning

The internal transcribed spacer (ITS) region, encompassing rRNAs, was amplified from the genomic DNA of *K. mikimotoi* using PCR with a pair of common primers, 18ScomF-3end and com28SR1 (the primer sequences are presented in Supplementary Table 1), as previously reported (Wang et al., 2014). Subsequently, the amplified ITS region containing A-overhangs at 3'-ends was sub-cloned into the T-Vector pMD^TM^19 (Takara) between T-overhangs at the cloning site, according to the manufacturer's instructions. The complete sequence of the vector and the ITS region (highlighted in yellow) is shown in Supplementary Figure 1.

### 2.5. *In vitro* digestion of ITS using LbCas12a

For the *in vitro* digestion, 1 μl of EnGen^®^ Lba Cas12a (1 μM) and 1 μl of crRNA (10 ng/μl) were mixed with 2 μl of 10 × NEBuffer^TM^ r2.1. Then, 150 ng of PCR-amplified ITS region of *K. mikimotoi* was added to the mixture, followed by incubation at 37°C for 1 h. The digestion products were separated by loading onto 1.5 % agarose horizontal gel in 1 × TAE buffer and subjected to electrophoresis at 100 volts for 30 min. The gel was visualized using a UV transilluminator.

### 2.6. LbCas12a-based reporter detection assay

Single-stranded decorated DNAs (5'-3': TTATT, ssDNA) were added to the LbCas12a/crRNA digestion reaction system (1 μl of EnGen^®^ Lba Cas12a (1 μM), 1 μl of crRNA (10 ng/μl), 2 μl of 10 × NEBuffer^TM^ r2.1 and plasmid, genomic DNA, or 2 μl of 20 μl RPA products). For LbCas12a-5'-FAM/3'-BHQ (FQ) reporter detection, 6.25 μM of FQ decorated ssDNAs were added. The reaction was performed at 37°C for 1 h, with fluorescence signal acquisition at 1-min intervals by Bio-Rad CFX96 Touch Real-time PCR Detection System. The endpoint fluorescent product was detected by a UV transilluminator or LED blue light. For LbCas12a-5'-FAM and 3'-Biotin (FB)-LFD reporter detection, 1 μM of FB decorated ssDNAs was added. After incubation at 37°C for 1 h, the final reaction products were transferred to a 2 ml centrifuge tube containing 100 μl of HybriDetect assay buffer (TwistDx^TM^) and incubated at room temperature for 2 min. The resulting mixture was then applied to a lateral flow strip (Milenia HybriDetect 1, TwistDx^TM^) and allowed to develop a colored readout for 2 min.

### 2.7. RPA primer design, RPA reaction, RPA-LbCas12a-FQ reporter, and -LbaCas12a-FB-LFD detection

RPA primers were designed using Primer3 (https://primer3.ut.ee). The specificity of the primers was evaluated by Primer-BLAST (https://www.ncbi.nlm.nih.gov/tools/primer-blast/index.cgi?LINK_LOC=BlastHome). The primer length ranged from 25 to 35 nucleotides, with melting temperatures of ~54 to 67°C.

The RPA reaction was performed following the protocol provided in the RAA kit (Hangzhou Zhongce Bio-Sci&Tech Co. Ltd). In brief, each lyophilized enzyme tube was reconstituted by 41.5 μl of buffer A and 2 μl of forward and reverse primers (10 μM). The reconstituted enzyme (18 μl) was distributed into PCR strip tubes. Then, 1 μl of each DNA sample was added to the reaction tubes, and 1 μl of DNase-free water was used as a negative control. Finally, 1 μl of buffer B was added to activate the reaction. After a brief vortex and quick centrifugation, the reaction was incubated at 37°C for 10 min.

For electrophoresis analysis, RPA products were extracted by phenol: chloroform: isoamyl alcohol (25:24:1), and the supernatants were loaded onto a 2 % agarose gel for visualization.

For RPA-LbCas12a-FQ reporter or -LbCas12a-FB-LFD detection, the RPA reaction was performed at 37°C for 10 min, followed by LbCas12a-FQ reporter or LbCas12a-FB LFD detection as described above.

## 3. Results

### 3.1. LbaCas12a cleaves ITS region of *K. mikimotoi* with specific crRNAs *in vitro*

**Figure 2:**
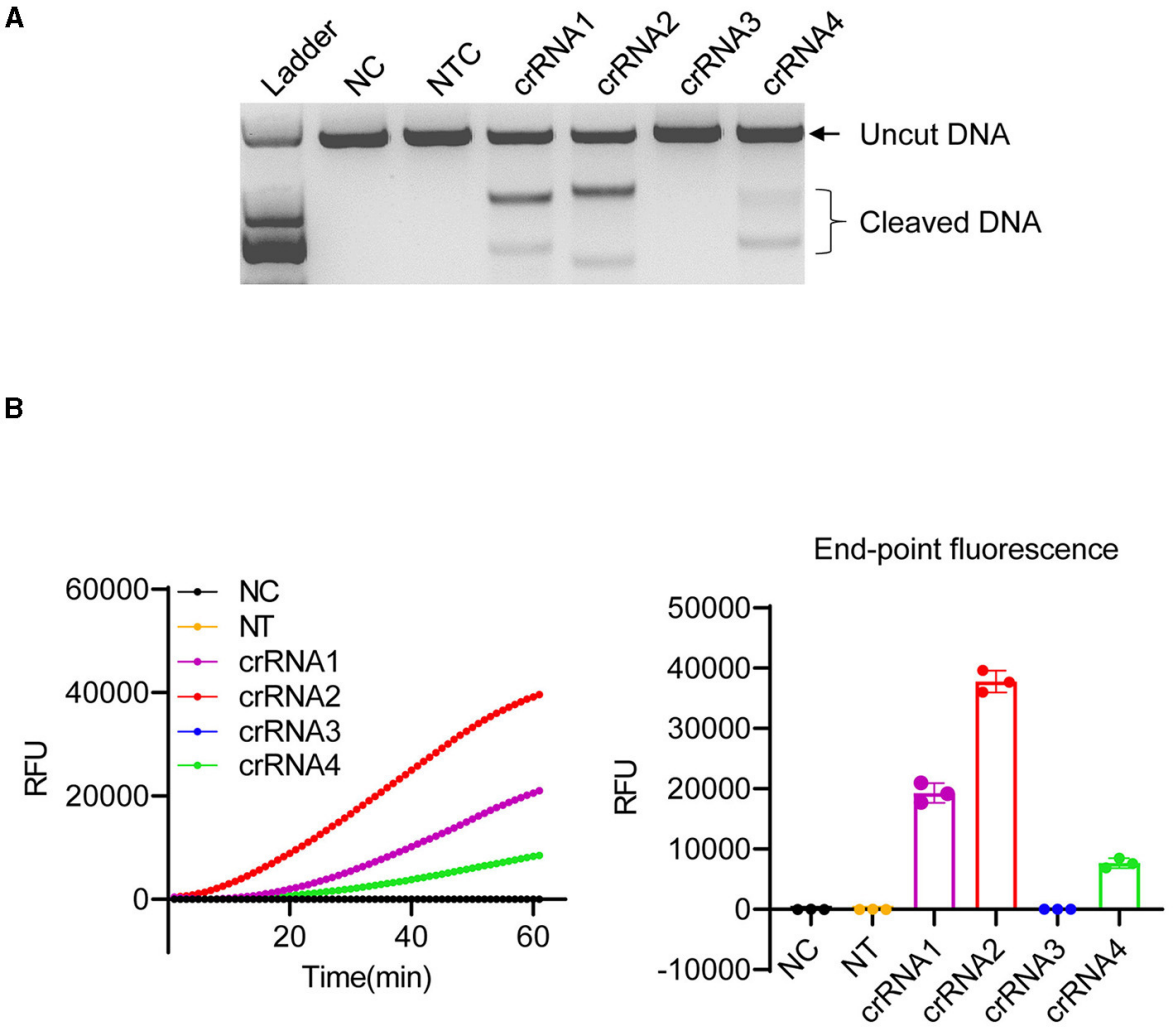
Optimization of LbCas12a cleavage *in vitro*. **(A)** *In vitro* digestion of 150 ng PCR product amplified from ITS of *K. mikimotoi*. Ladder: DL2,000 (Takara); NC, negative control (nuclease-free water); NTC, non-target control (targeting *Karlodinium veneficum*). **(B)** Cleavage activity of LbCas12a detected by a real-time PCR machine. Error bars denote SD (*n* = 3).

The ITS of the ribosomal RNA cistron is known for its variability (Lin et al., 2009) and its potential to serve as a unique species-specific DNA barcode for most dinoflagellates (Wang et al., 2014). In this study, we used the ITS region (Supplementary Figure 1) as the detection marker of *K. mikimotoi*. To determine whether LbCas12a exhibits activity toward the selected ITS region with specific crRNAs, a 1,580 bp fragment of the ITS region was amplified using a pair of common primers (Wang et al., 2014) (Supplementary Table 1), for *in vitro* cleavage assay (Figure 2A). As LbCas12a only requires a short CRISPR RNA (crRNA) for recognition and cleavage of the target DNA, along with a short T-rich protospacer-adjacent motif (5'-TTTV-3', PAM, V = A/G/C) for efficient cleavage (Zetsche et al., 2015), four crRNAs (crRNA1, 2, 3, and 4) were synthesized, specifically targeting the opposite strand of the ITS variable region with the PAM sequence of *K. mikimotoi* (accession number: KF998563) (Supplementary Figure 2 and Supplementary Table 1). The non-target control crRNA (NTC, Supplementary Table 1) targeting *Karlodinium veneficum* (*K. veneficum*) showed no cleavage activity. However, crRNAs targeting *K. mikimotoi*, except for crRNA3, cleaved the target region with various activities (Figures 2A, B). The sizes of the cleaved products corresponded to the protospacer region (Figure 2A), indicating that the PCR product was cut into two fragments. Notably, in addition to Cas12a's double-stranded DNA (dsDNA) cleavage activity (Figure 2A), it also exhibited various “collateral activities” toward ssDNAs (Figure 2B, Supplementary Figure 3). The presence of two adjacent mismatches in crRNA3 abolished the cleavage activity of LbCas12a toward the target sequence (Figures 2, Supplementary Figures 2, 3), and crRNA1 exhibited lower cleavage activity compared with crRNA2 (Figure 2B), which is consistent with the tolerance of LbCas12a to mismatch at position 8 (Kleinstiver et al., 2016) and mismatched nucleotides at PAM or protospacer can lead to compensated cleavage activity (Figures 2A, B) (Kim et al., 2016; Kleinstiver et al., 2016; Chen et al., 2018). Furthermore, crRNA2 and 4, which matched the target sequence, also exhibited various activities (Figure 2B and Supplementary Figure 2), suggesting the need for optimization of crRNAs. In conclusion, our results demonstrated that LbCas12a efficiently cleaved target algal DNA fragments in a crRNA-dependent manner.

### 3.2. RPA enhances the sensitivity of LbCas12a-based detection

**Figure 3:**
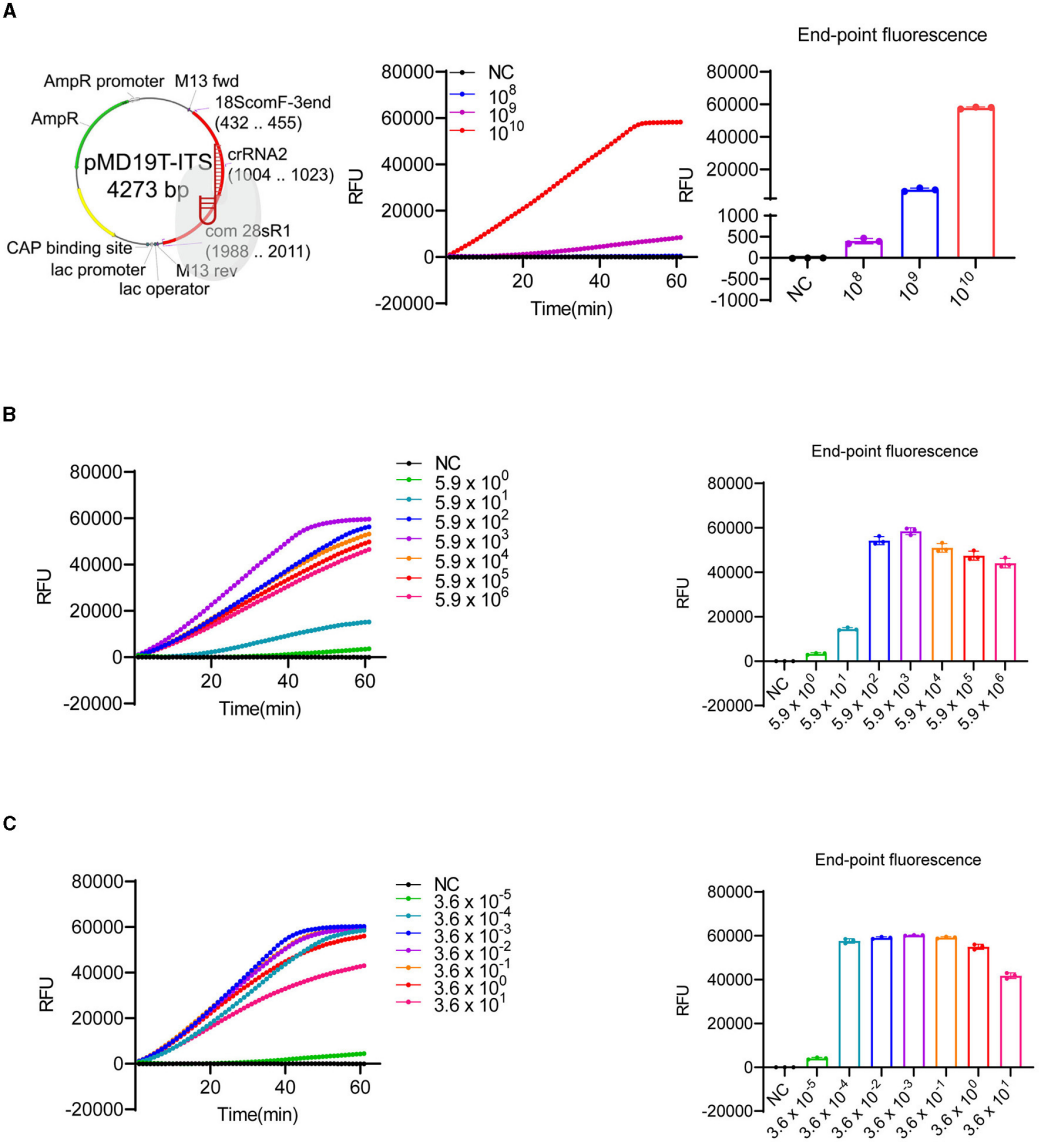
Sensitivity test of LbCas12a-based detection. **(A)** The plasmid map with ITS insertion, indicating digestion by LbCas12a, was generated by SnapGene and created with BioRender (https://www.biorender.com/). LbCas12a-FQ reporter assays were performed using 10-fold serial dilutions of plasmids containing ITS of *K. mikimotoi*. The copy number of the plasmids ranged from 5.93 × 10^10^ to 5.93 × 10^0^ copies/μl (only dilutions showing signals were included in the figure). **(B)** LbCas12a-FQ reporter assays were conducted with pre-amplified substrates by RPA using 10-fold serial dilutions of plasmids containing ITS of *K. mikimotoi*. The copy number of the plasmids ranged from 5.93 × 10^6^ to 5.93 × 10^0^ copies/μl. **(C)** LbCas12a-FQ reporter assays were conducted with pre-amplified substrates by RPA using a 10-fold serial dilution of *K. mikimotoi* genomic DNA. The concentrations of genomic DNA ranged from 3.6 × 10^1^ to 3.6 × 10^−5^ ng/μl. NC, negative control (nuclease-free water), error bars denote SD (*n* = 3).

The timely monitoring of HABs is crucial for minimizing economic losses and protecting marine ecosystems and public health (Sun et al., 2019). Therefore, detecting HABs at low densities is essential. In this study, we first evaluated the sensitivity of our LbCas12a-FQ or -FB-LFD reporter detection system. We sub-cloned the amplified fragment (Supplementary Figure 1, highlighted sequence) into a T-Vector and prepared 10-fold serial dilutions of the plasmid, ranging from 5.9 to 5.9 × 10^10^ copies/μl, with nuclease-free water as the negative control. Since crRNA2 exhibited the highest activity (Figure 2), it was selected for subsequent experiments. We detected the plasmid only when its concentration was above 5.9 × 10^8^ copies/μl (equivalent to 0.98 nM) (Figure 3A), consistent with the low collateral activity of LbCas12a (Chen et al., 2018; Gootenberg et al., 2018; Kellner et al., 2019, 2020). To enhance the sensitivity, we employed RPA, which is a field-portable method that operates at a lower reaction temperature (Chen et al., 2018; Gootenberg et al., 2018; Kellner et al., 2019, 2020), to pre-amplify the target DNA. After comparing the specificity (data not shown) and yield of different RPA primer pairs (Supplementary Figure 4A), we selected Km_RPA1 (Supplementary Table 1) as the primer set for pre-amplification. With the combination of crRNA2 and Km_RPA1, we achieved increased sensitivity, detecting fewer than 6 copies/μL (equivalent to 9.8 aM) of the plasmid carrying the target region and <10^−5^ ng/μL genomic DNA of *K. mikimotoi* (Figures 3B, C). These results demonstrated that the sensitivity of LbCas12a-based detection was substantially enhanced by pre-amplification with RPA, enabling the early detection of *K. mikimotoi*.

### 3.3. LbCas12a with specific crRNAs ensures the specificity of the detection

**Figure 4:**
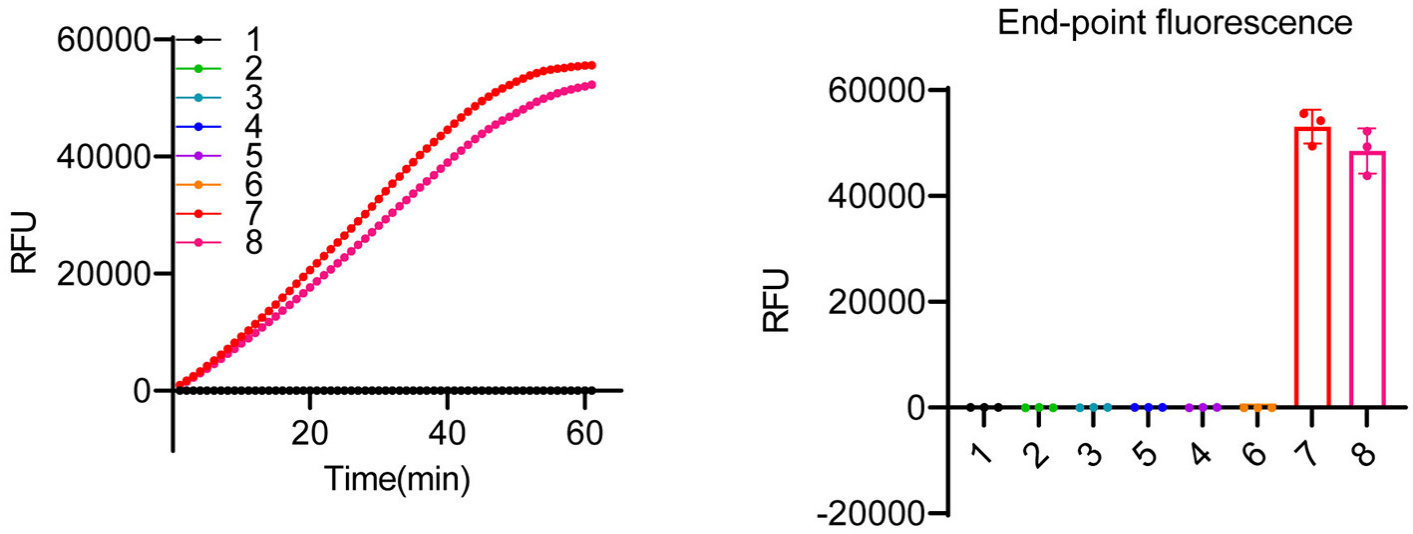
Specificity test of LbCas12a-based detection system. LbCas12a-FQ reporter assays were performed using genomic DNAs from various algal species. The samples included nuclease-free water (1), *Heterocapsa* sp. (2), *Amphidinium carterae* (3), *Prorocentrum triestinum* (4), *Isochrysis galbana* (5), *Dicrateria zhanjiangensis* (6), *K. mikimotoi* A (7), and *K. mikimotoi* B (8). Error bars denote SD (*n* = 3).

Although some undesired non-specific amplification was observed in the RPA reactions (Supplementary Figure 4A), the specificity of the detection can be ensured by crRNAs of LbCas12a (Kellner et al., 2019, 2020). As shown in Figure 2, LbCas12a only cleaved ITS fragments from *K. mikimotoi* when a crRNA specifically targeting *K. mikimotoi* was used. In contrast, crRNA targeting *K. veneficum* had no cleavage effect on *K. mikimotoi* ITS. To further demonstrate the specificity of LbCas12a-crRNA, particularly with RPA pre-amplification, we applied LbCas12a-FQ reporter and -FB-LFD detection to six species of algae, including those that co-occur with *K. mikimotoi* during bloom, such as *Heterocapsa* sp. and *Prorocentrum triestinum*, as well as *Isochrysis galbana*, which does not cause harmful blooms, and *Dicrateria zhanjiangensis*, which is rarely found in red tides along the Fujian coast. In comparison with the negative control and other species, only two strains of *K. mikimotoi* (A and B) could be detected (Figure 4 and Supplementary Figures 4B, C), and the RPA pre-amplified non-specific products (Supplementary Figure 4A) were not detected (Figure 4). These findings indicated that crRNAs of LbCas12a were sufficient for ensuring the specificity of the detection, despite the possibility of undesired non-specific amplification of RPA.

### 3.4. CFP-based purification coupled RPA-LbCas12a detection further simplifies the detection system

**Figure 5:**
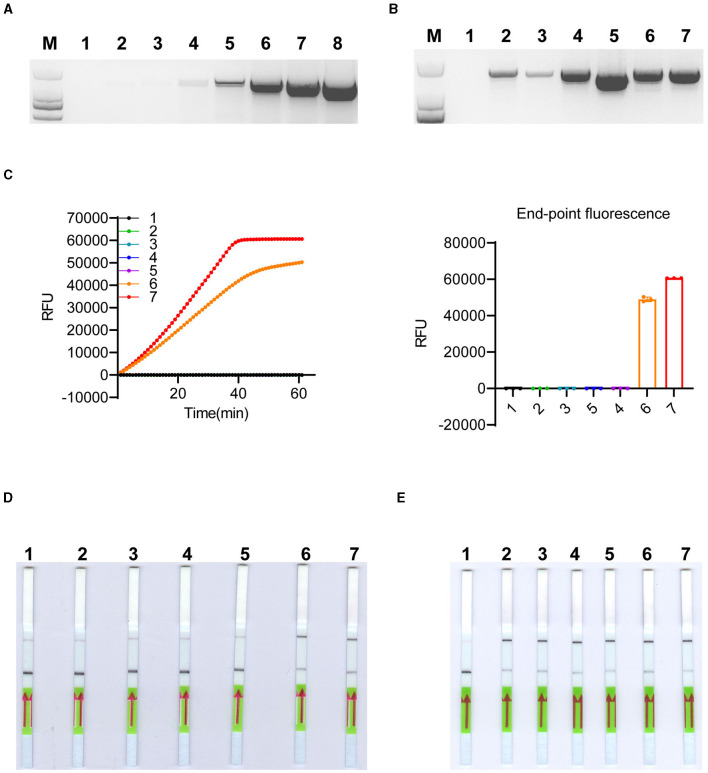
CFP-based genomic DNA extraction and LbCas12a-based detection. **(A)** Conventional PCR amplification of the ITS region was performed using genomic DNA extracted by CFP from a 10-fold serial dilution of *K. mikimotoi* cells. The cell concentration ranged from 10^0^ to 10^6^ cells/ml. Samples 2–8 represent the dilutions with cell concentrations ranging from 10^0^ to 10^6^ cells/ml, while sample 1 corresponds to nuclease-free water. M: DL2,000 DNA ladder. **(B)** Conventional PCR amplification of ITS region was performed using genomic DNA extracted by CFP from 1,000 algal cells of different species, including *K. veneficum, Prorocentrum triestinum, Karenia papilionacea, Alexandrium pacificum, K. mikimotoi* A, and *K. mikimotoi* B, respectively. Nuclease-free water was labeled as 1 and used as a negative control. M: DL2,000 DNA ladder. **(C)** LbCas12a-FQ reporter assays were conducted using substrates obtained through RPA pre-amplification with DNA extracted by CFP from the samples, as shown in **(B)**. Error bars denote SD (*n* = 3). **(D)** LbCas12-FB-LFD was performed with substrates obtained through RPA pre-amplification with DNA extracted by CFP from the samples, as shown in **(B)**. **(E)** LbCas12-FB-LFD was performed to detect 10-fold serially diluted spiked *K. mikimotoi* samples with cell concentrations ranging from 10^3^ to 10^−2^ cells/ml. The negative control (1) was nuclease-free water, while samples (2–7) represented cell concentrations ranging from 10^−2^ to 10^3^ cells/ml.

The extraction and purification of algal genomic DNA can be a time-consuming and labor-intensive process, and obtaining genomic DNA quickly and economically is crucial for diagnosis, particularly in field-based testing (Zou et al., 2017). The instrument-free and low-cost untreated CFP has been used for the extraction of DNA and RNA from plants, animals, and microbes (Zou et al., 2017). We tested whether CFP could also be used for microalgae DNA extraction. Genomic DNA from serially diluted *K. mikimotoi* algal cells, ranging from 10^6^ cells/ml to 1 cell/ml, was extracted using CFP, and the eluted genomic DNA was directly used for PCR amplification of the target fragment (~1500 bp), showing effective amplification across the gradient (Figure 5A). Additionally, we tested CFP purification on other algal cells with varying shell thicknesses, which can affect the efficiency of DNA extraction using cetyl/hexadecyl trimethyl ammonium bromide in 0.7 M NaCl solution (Yuan et al., 2015). The results demonstrated successful amplification of the target fragment from all selected cells (Figure 5B). Considering that the extracted DNAs were easily amplified by conventional PCR, we further investigated their suitability for RPA pre-amplification and detection using LbCas12a reporters. The genomic DNAs extracted from Figure 5B were subjected to RPA-LbCas12a-FQ or -FB-LFD detection. As expected, only *K. mikimotoi* species can be detected using RPA-LbCas12a-FQ and RPA-LbCas12a-FB-LFD (Figures 5C, D and Supplementary Figures 5A, B). To determine the limit of detection, we applied CFP-based genomic DNA extraction with RPA-LbCas12a reporters to spiked *K. mikimotoi* cells ranging from 10^3^ to 10^−2^ cells/ml. All diluents were successfully detected using CFP-coupled RPA-LbCas12a-FQ or -FB-LFD (Figure 5E and Supplementary Figures 5C, D), indicating that the detection can reach the single-cell level. These findings collectively suggested that genomic DNA from algal cells can be purified using CFP and subsequently identified using RPA-LbCas12a-FB-LFD, facilitating field-deployed detection.

## 4. Discussion

Despite their potential, Cas12a-based diagnostics have not been fully utilized in the identification of HABs. In this study, we applied the DETECTR (Chen et al., 2018) for the detection of *K. mikimotoi*, one of the HAB-forming dinoflagellates (Figure 1). The method relies on the enzymatic activities of LbCas12a toward RPA pre-amplified specific dsDNA (cis cleavage) and non-specific ssDNA (trans cleavage) (Zetsche et al., 2015; Chen et al., 2018).

Selecting the target gene for probing or priming is a crucial step in the development of a molecular detection assay. Commonly used target genes for discriminating microalgae species include small subunit rDNA (Zhen et al., 2008, 2009), large subunit rDNA (Guillou et al., 2002; Haywood et al., 2007; Bowers et al., 2017; Zhang et al., 2018), and ITS (Huang et al., 2017a,b, 2020; Engesmo et al., 2018; Zhang et al., 2019; Wang et al., 2020; Han et al., 2022). The ITS regions have been widely used for species discrimination in microalgae (Connell, 2002), including *K. veneficum* (Huang et al., 2017a; Fu et al., 2019) and *K. mikimotoi* (Yuan et al., 2012; Zhang et al., 2019; Huang et al., 2020; Wang et al., 2020; Han et al., 2022). For most dinoflagellate genera, the ITS region is considered an effective species identifier (Stern et al., 2012). In a previous study, we demonstrated the suitability of the ITS region as a DNA barcode for resolving the “*Alexandrium tamarense* species complex”, which is challenging to identify (Wang et al., 2014). Therefore, in this study, we selected the ITS region of *K. mikimotoi* as the detection target.

Most of the molecular techniques currently used for the detection of *K. mikimotoi* rely on the direct detection of amplified products, such as qPCR (Yuan et al., 2012; Hu et al., 2022) and LAMP-LFD (Han et al., 2022). The specificity of these methods depends primarily on the amplification primers that can tolerate a certain number of mismatches, although the use of additional probes can help improve specificity. However, the LAMP-LFD requires the incorporation of specific fluorophore-decorated probes into the amplification reaction for visualization, which increases the cost of detection as these specific probes cannot be used with other species. The tolerance of non-specific amplification also exists in RPA pre-amplified products (Supplementary Figure 4A). In contrast, Cas12a-based methods detect the amplified products by recognizing dsDNA guided by crRNAs and releasing the visualizing signals using non-specific fluorophore-decorated ssDNAs, which can be shared with other species. The specificity of Cas12a-based detection can reach to single-base nucleotide level, as mismatches in the PAM-adjacent “seed region” of crRNAs inhibit the Cas12a activity (Chen et al., 2018; Zhang et al., 2020) (Figures 2 and Supplementary Figure 2). We also observed that the activity of Cas12a varied with different crRNAs, even in the absence of any mismatches (crRNA2 and crRNA4), suggesting the importance of experimentally testing crRNA activity, although tools such as CRISPRscan, CHOPCHOP, and Benchling can be used for potential crRNAs design. It is worth noting that the requirement for a specific PAM limits the selection of target regions, and alternative Cas12a variants that recognize different PAMs should be considered (Liu et al., 2021). Additionally, for unknown strains of *K. mikimotoi*, confirmation with PCR using common primers for the ITS region (see Supplementary Table 1) should be performed as the designed crRNAs are based on the known ITS sequences.

In addition to the specificity of the detection method, sensitivity is also a critical concern. More than 10^4^ cells/ml of *K. mikimotoi* can result in significant fish mortality in aquaculture regions (Wang et al., 2001), and as few as 20 cells/ml can cause 50 % mortality of rotifers in 24 h and reduce the survival rate of scallop eyespot larva in 96 h (Sun et al., 2010). These findings highlight the potential harm of *K. mikimotoi*, a toxin-producing HAB, even at low densities in field samples (Li et al., 2019b). Therefore, it is crucial to develop a sensitive method capable of detecting *K. mikimotoi* at low densities to provide early warning of potential harm. To achieve increased sensitivity, the pre-amplification of the target is necessary. Conventional PCR and isothermal amplification methods, such as RPA (Piepenburg et al., 2006), LAMP (Tomita et al., 2008), and helicase-dependent amplification (Vincent et al., 2004), can be employed to amplify the target gene for DNA detection. However, PCR requires multiple thermal cycles in a thermal cycler for amplification. LAMP typically operates at a constant temperature range of 60–65°C, which can reduce the non-specific effects, but the complexity of primer design and the high-temperature limit its field applications. In contrast to PCR and LAMP, RPA can be performed at 37–42°C, making it more suitable for field deployment. Although RPA carries the risk of non-specific amplification, which requires the screening of multiple pairs of primers or incorporating probes to enhance the detection stringency as an alternative approach, the specificity of RPA can be further ensured by crRNA of LbCas12a (Kellner et al., 2019, 2020). In this study, we utilized RPA, one of the isothermal amplification methods, to pre-amplify the ITS region fragment, resulting in an increased sensitivity of the Cas12a-based detection from 0.98 nM to 9.8 aM (Figure 3), which is nearly equivalent to the detection of a single cell.

Finally, we simplified and expanded the application of CFP-based DNA purification for *K. mikimotoi* (Zou et al., 2017). Traditional DNA purification methods for algal cells usually involve proteinase digestion in a lysis buffer (see Materials and methods section), which takes hours to 3 days, followed by DNA clean columns. Although this method yields high-purity DNA, it requires a centrifuge and DNA clean columns and is time-consuming. In contrast, CFP-based DNA purification offers a more convenient and cost-effective approach for the detection of *K. mikimotoi*, utilizing the dipstick extraction method that takes <30 s. The results of spiked samples demonstrated that CFP-based genomic DNA extraction can achieve a single-cell level detection with RPA-LbCas12a reporters (Figure 5E and Supplementary Figures 5C, D).

In summary, we successfully applied CRISPR/Cas12a for the detection of *K. mikimotoi*, a bloom-forming dinoflagellate. The utilization of CFP-based DNA purification in combination with LbCas12a-FB-LFD detection, operating at a reaction temperature of 37°C in <1 h, provides significant advantages for field deployment.

## Data availability statement

The raw data supporting the conclusions of this article will be made available by the authors, without undue reservation.

## Author contributions

LW and JC conceived the original ideas, designed the project, and wrote the manuscript with inputs from XC, FP, and GY. LW performed most of the experiments with participation from XC, FP, and GY. All authors contributed to the article and approved the submitted version.
