# Supplementary material for: Development of a rapid detection method for Karenia mikimotoi by using CRISPR-Cas12a
Source: Front Microbiol. 2023 Aug 7;14:1205765. doi: 10.3389/fmicb.2023.1205765 (PMC10440436; doi:10.3389/fmicb.2023.1205765)
Supplement: Supplementary file 1 [file Data_Sheet_1.pdf]

Supplementary Material

Development of a Rapid Detection Method for *Karenia mikimotoi* by Using CRISPR-Cas12a

Lu Wang[1]*, Xiaoyao Chen[2], Feifei Pan[2], Guangshan Yao[1], Jianming Chen[1]*

***Correspondence:**

Lu Wang: 607wanglu@mju.edu.cn and Jianming Chen: chenjm@mju.edu.cn

1 Supplementary Figures and Tables

1.1 Supplementary Figures

>pMD19-ITS

TCGCGCGTTTCGGTGATGACGGTGAAAACCTCTGACACATGCAGCTCCCGGAGACGGTC
ACAGCTTGTCTGTAAGCGGATGCCGGGAGCAGACAAGCCCGTCAGGGCGCGTCAGCGGG
TGTTGGCGGGTGTCGGGGCTGGCTTAACTATGCGGCATCAGAGCAGATTGTACTGAGAGT
GCACCATATGCGGTGTGAAATACCGCACAGATGCGTAAGGAGAAAATACCGCATCAGGCG
CCATTCGCCATTCAGGCTGCGCAACTGTTGGGAAGGGCGATCGGTGCGGGCCTCTTCGCT
ATTACGCCAGCTGGCGAAAGGGGGATGTGCTGCAAGGCGATTAAGTTGGGTAACGCCAG
GGTTTTCCCAGTCACGACGTTGTAAAACGACGGCCAGTGAATTCGAGCTCGGTACCCGGG
GATCCTCTAGAGATGTCGTAACAAGGTTTCCGTAGGTGAACCTGCGGAAGGATCATTCAC
ACGCATCCAACTTGAATTCATTGTGAACCATCTTCTGTGAGTTGTGCTCTTGGCTGGAGAC
AACTTGTCTACAACCTTGTGCAGAATCTCGCGGGTAGTTCGGGTGATATTGGACCGTCTTC
CTCTATTGTCTGGCTACCATTGTTGTCTGACCAATGTCTTGCATGATGACCTATCTTTTTAA
GATGTGGGTGACCACATGTTTGATTCACATACAACTTTCAGCGACGGATGTCTCGGCTCG
AACAACGATGAAGGGCGCAGCGAAGTGTGATAAGCATTGTGAATTGCAGAATTCCGTGA
ACCAATAGGGATTTGAACGTATACTGCGCTTTCGGGATATCCCTGAAAGCATGCCTGCTTC
AGTGTCAATATTCTCTCATGCCACTGTCATCTTGTCATGTGCTTTGCACATGCAACTGACA
GCAGTGTGTGTCTGTGCATTAAGGTGCTCTTCCTGCCCCTGATGCATTGAATCCATGGGTT
TCGTGCTCTGTCAGCAACTTGATAAACACCTTGTGCTTTGTGTGTAACCTGTTGCTTTGTC
TGTTGCACGTCAACCTGTCCTCCTGTCTGTCACTTCATTTGTGGCTTCTTTCCATGACATG
AAGTTAGGTAAGCAAACCCGCTGAATTTAAGCATATAAGTAAGCGGAGGATAAGAAACTA
AATAGGATTCCCTCAGTAATGGCGAATGAACAGGGATAAGCTCAGCATGGAAATTGGGGC
CCTCGGCCTTGAATTGTAGTCTTGAGATGTGTTACCAACGGAGGCGCAGATGTAAGCCTC
TTGGAAAAGAGCGTCAGGGAGGGTGAGAGTCCCGTATGTCATCTGCAGTTCTCTGTGCAC
GGTGCATGTTCTAAGAGTCACGTTCCTCGGGATTGGAGCGCAAATTGGGTGGTAAATTTC
ATCTAAAGCTAAATATTGGTTCGAGACCGATAGCAAACAAGTACCATGAGGGAAAGGTGA
AAAGGACTTTGAAAAGAGAGTTAAAAGTGCCTGAAATTGCTGAAAGGGAAGCGAATGG
AACCAGTTGTTCTTGGTGAGTATTGGTGTTGTCTAAAGTGATGGCTTGCCACTTCAACGCA

AGTGTGGTGGCAGGTTTTGATCTGGATGCGATACTGCTTCTCGCCTTGCATGTCAACGTCA
GTTCATAATTGAGGAAAACTCTAAGGACATGGTAATTCGCTTCCGAGTGACTGAATGTCCT
CAGTTGAACTCATTTTTGAACTGCTCTCTGTGTGTCTGGTAGCACTGCTTCATGTGCTTGC
CTGCGATCTTCTGCTCTGCATGAAGGTTGTTGGTGCCAGGAGCATGTCCTTGACATTAGAA
CGATGACGAAATGGTTTTATTCGACCCGTCTTGAAACACGGACCAAGGAGTCTAACATAT
GTGCAAGTTCACGGGCGGAAAACCTGCTTGCGCAATGAAAGTGACTGCTGGGATTTTT
GCACCAGCAACCGACCAATCAATTGTGAGAGGTTTGAGTATGAGCATATCTGTTAGGACC
CGAAAGATGGTGAACTATGCGTGAtATCGTCGACCTGCAGGCATGCAAGCTTGGCGTAATC
ATGGTCATAGCTGTTTCCTGTGTGAAATTGTTATCCGCTCACAATTCCACACAACATACGA
GCCGGAAGCATAAAGTGTAAAGCCTGGGGTGCCTAATGAGTGAGCTAACTCACATTAATT
GCGTTGCGCTCACTGCCCGCTTTCCAGTCGGGAAACCTGTCGTGCCAGCTGCATTAATGA
ATCGGCCAACGCGCGGGGAGAGGCGGTTTGCGTATTGGGCGCTCTTCCGCTTCCTCGCTC
ACTGACTCGCTGCGCTCGGTCGTTCGGCTGCGGCGAGCGGTATCAGCTCACTCAAAGGC
GGTAATACGGTTATCCACAGAATCAGGGGATAACGCAGGAAAGAACATGTGAGCAAAAG
GCCAGCAAAAGGCCAGGAACCGTAAAAAGGCCGCGTTGCTGGCGTTTTTCCATAGGCTC
CGCCCCCCTGACGAGCATCACAAAAATCGACGCTCAAGTCAGAGGTGGCGAAACCCGAC
AGGACTATAAAGATACCAGGCGTTTCCCCCTGGAAGCTCCCTCGTGCGCTCTCCTGTTCC
GACCCTGCCGCTTACCGGATACCTGTCCGCCTTTCTCCCTTCGGGAAGCGTGGCGCTTTCT
CATAGCTCACGCTGTAGGTATCTCAGTTCGGTGTAGGTCGTTCGCTCCAAGCTGGGCTGTG
TGCACGAACCCCCCGTTCAGCCCGACCGCTGCGCCTTATCCGGTAACTATCGTCTTGAGTC
CAACCCGGTAAGACACGACTTATCGCCACTGGCAGCAGCCACTGGTAACAGGATTAGCA
GAGCGAGGTATGTAGGCGGTGCTACAGAGTTCTTGAAGTGGTGGCCTAACTACGGCTACA
CTAGAAGAACAGTATTTGGTATCTGCGCTCTGCTGAAGCCAGTTACCTTCGGAAAAAGAG
TTGGTAGCTCTTGATCCGGCAAACAAACCACCGCTGGTAGCGGTGGTTTTTTTGTTTGCA
AGCAGCAGATTACGCGCAGAAAAAAAGGATCTCAAGAAGATCCTTTGATCTTTTCTACGG
GGTCTGACGCTCAGTGGAACGAAAACTCACGTTAAGGGATTTTGGTCATGAGATTATCAA
AAAGGATCTTCACCTAGATCCTTTTAAATTAAAAATGAAGTTTTAAATCAATCTAAAGTATA
TATGAGTAAACTTGGTCTGACAGTTACCAATGCTTAATCAGTGAGGCACCTATCTCAGCGA
TCTGTCTATTTCGTTCATCCATAGTTGCCTGACTCCCCGTCGTGTAGATAACTACGATACGG
GAGGGCTTACCATCTGGCCCCAGTGCTGCAATGATACCGCGAGACCCACGCTCACCGGCT
CCAGATTTATCAGCAATAAACCAGCCAGCCGGAAGGGCCGAGCGCAGAAGTGGTCCTGC
AACTTTATCCGCCTCCATCCAGTCTATTAATTGTTGCCGGGAAGCTAGAGTAAGTAGTTCG
CCAGTTAATAGTTTGCGCAACGTTGTTGCCATTGCTACAGGCATCGTGGTGTCACGCTCGT
CGTTTGGTATGGCTTCATTCAGCTCCGGTTCCCAACGATCAAGGCGAGTTACATGATCCCC
CATGTTGTGCAAAAAAGCGGTTAGCTCCTTCGGTCCTCCGATCGTTGTCAGAAGTAAGTT
GGCCGCAGTGTTATCACTCATGGTTATGGCAGCACTGCATAATTCTCTTACTGTCATGCCAT
CCGTAAGATGCTTTTCTGTGACTGGTGAGTACTCAACCAAGTCATTCTGAGAATAGTGTAT
GCGGCGACCGAGTTGCTCTTGCCCGGCGTCAATACGGGATAATACCGCGCCACATAGCAG
AACTTTAAAAGTGCTCATCATTGGAAAACGTTCTTCGGGGCGAAAACTCTCAAGGATCTT
ACCGCTGTTGAGATCCAGTTCGATGTAACCCACTCGTGCACCCAACTGATCTTCAGCATCT
TTTACTTTCACCAGCGTTTCTGGGTGAGCAAAAACAGGAAGGCAAAATGCCGCAAAAAA
GGGAATAAGGGCGACACGGAAATGTTGAATACTCATACTCTTCCTTTTTCAATATTATTGA
AGCATTTATCAGGGTTATTGTCTCATGAGCGGATACATATTTGAATGTATTTAGAAAAATAA
ACAAATAGGGGTTCCGCGCACATTTCCCCGAAAAGTGCCACCTGACGTCTAAGAAACCAT
TATTATCATGACATTAACCTATAAAAATAGGCGTATCACGAGGCCCTTTCGTC

Supplementary Figure 1. The sequence of T-Vector pMD^{TM}19 and ITS region of *K. mikimotoi*

The ITS region was highlighted in yellow and subcloned into the vector between two “T” in red font.

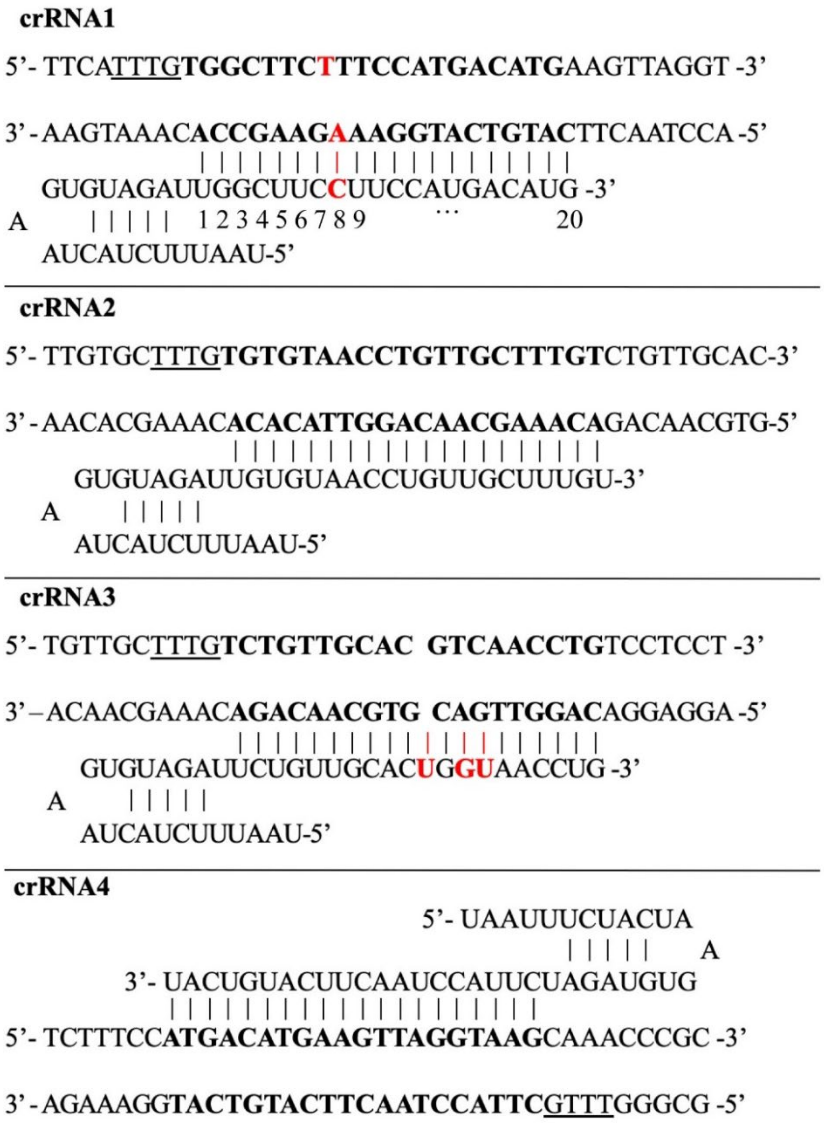

Supplementary Figure 2. DNA sequences of crRNA targets and corresponding crRNA sequences

The underlined sequence represents the PAM sequence, the sequence with numeric values on crRNA1 indicates a target-specific sequence of crRNA, while the other nucleotides in the sequence are the crRNA repeat. Mismatches between targets and crRNAs are in red font.

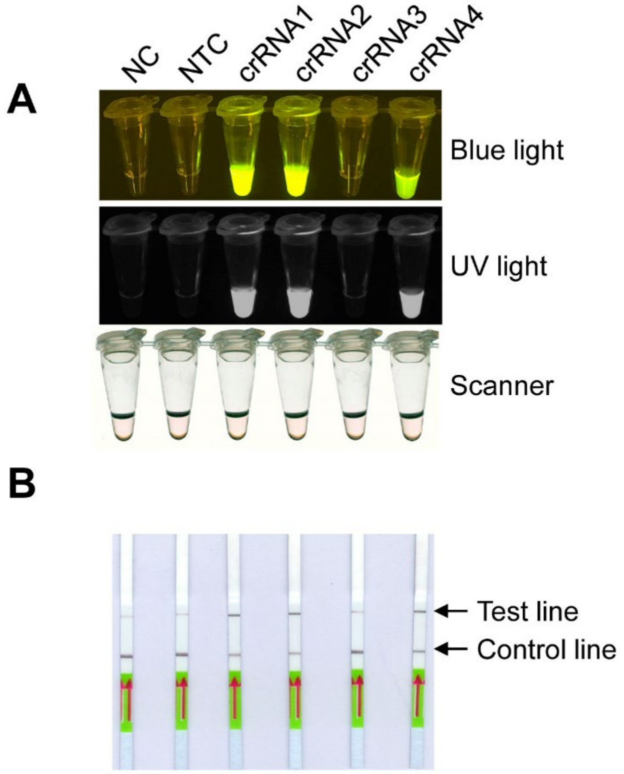

Supplementary Figure 3. Detection of LbCas12a cleavage activity in vitro

A. Endpoint fluorescent product generated from LbCas12a-FQ reactions was imaged by a smartphone camera under blue light, a UV transilluminator, and a scanner. The samples imaged corresponded to those in Figure 2B.

B. LbCas12a-FB-LFD detection of samples from Figure 2B.

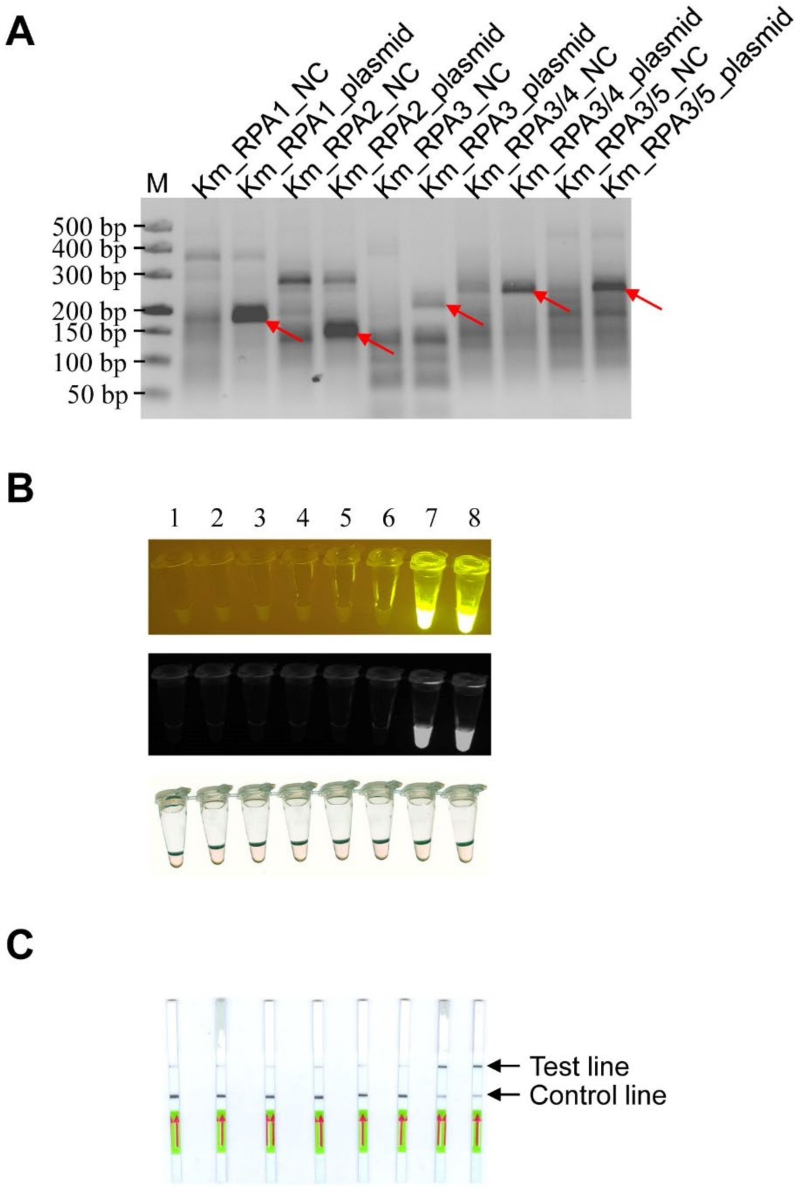

Supplementary Figure 4. Specificity of the RPA-LbCas12a-based detection system

A. Amplification of the ITS region of *K. mikimotoi* was performed using different RPA primer sets. NC: nuclease-free water, plasmid: pMD19-ITS, M: 500 bp DNA ladder, anticipated bands were annotated by red arrows.

B. Endpoint fluorescent product was imaged using a smartphone camera under blue light, a UV transilluminator, and a scanner. The samples corresponded to those shown in Figure 4.

C. LbCas12a-FB-LFD detection of samples from Figure 4.

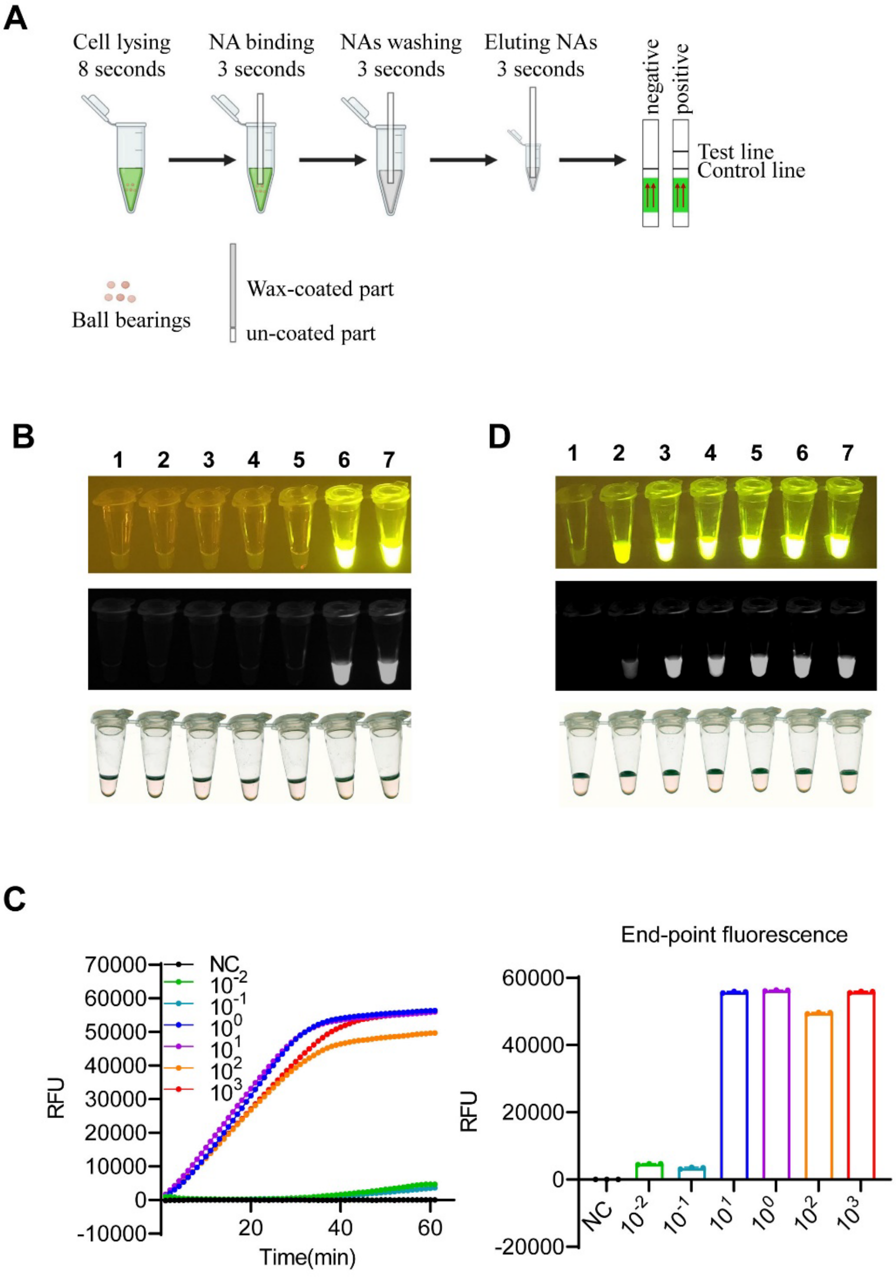

Supplementary Figure 5. Detection of CFP-extracted DNA using LbCas12a-based reporters

A. Flowchart of CFP-based genomic DNA extraction and LbCas12a-FB-LFD assays. Created with BioRender (https://www.biorender.com/). NA: nucleic acids

B. Endpoint fluorescent product was imaged using a smartphone camera under blue light, a UV transilluminator, and a scanner. The samples corresponded to those in Figure 5B-D.

C. LbCas12a-FQ reporter assays were performed using substrates obtained through RPA pre-amplification with genomic DNA extracted by CFP from 10-fold serial dilutions of *K. mikimotoi* cells ranging from 10^3 to 10^{-2} cells/mL. NC: negative control (nuclease-free water); error bars denote SD (n = 3).

D. Endpoint fluorescent product was imaged by a smartphone camera under blue light, a UV transilluminator, and a scanner. The imaged samples corresponded to those shown in Figure 5E.

1.2 Supplementary Tables

Supplementary Table 1. Sequence information for all primers used in the study

The sequence of PCR primers, guide RNAs, RPA primers, and decorated ssDNAs.

**common primers for ITS region**	**Sequence (5'→3')**
18ScomF-3end	5'-GTCGTAACAAGGTTTCCGTAGGTG-3'
com28sR1	5'-TCACGCATAGTTCACCATCTTTCG-3'

**guide RNA**	**Sequence (5'→3')**
NTC	UAAUUUCUACUAAGUGUAGAUUGAGCUCUUGCGCGGGGGUU
crRNA1	UAAUUUCUACUAAGUGUAGAUUGGCUUCCUUCCAUGACAUG
crRNA2	UAAUUUCUACUAAGUGUAGAUUGUGUAACCUGUUGCUUUGU
crRNA3	UAAUUUCUACUAAGUGUAGAUUCUGUUGCACUGGUAACCUG
crRNA4	UAAUUUCUACUAAGUGUAGAUCUUACCUAACUUCAUGUCAU

**RPA primer**	**Sequence (5'→3')**
Km_RPA1_fw	CTCTGTCAGCAACTTGATAAACACC
Km_RPA1_rs	GTTTCTTATCCTCCGCTTACTTATA
Km_RPA2_fw	CAGCAACTTGATAAACACCTTGTGC
Km_RPA2_rs	AAATTCAGCGGGTTTGCTTACCTAA
Km_RPA3_fw	AATCCATGGGTTTTGTGCTCTGTCAGCAACTT
Km_RPA3_rs	CTATTTAGTTTCTTATCCTCCGCTTACTTATA
Km_RPA4_rs	TGAGCTTATCCCTGTTCATTCGCCATTACTGA
Km_RPA5_rs	TCCATGCTGAGCTTATCCCTGTTCATTCGCCA

**ssDNA-reporter**	**Sequence (5'→3')**
FQ	5'/6-FAM/TTATT/BHQ/3'
FB	5'/6-FAM-TTATT/Biotin/3'
